# The role of music in promoting health and wellbeing: a systematic review and meta-analysis

**DOI:** 10.1093/eurpub/ckad063

**Published:** 2023-06-15

**Authors:** Erica Viola, Marco Martorana, Chiara Airoldi, Cristina Meini, Daniele Ceriotti, Marta De Vito, Damiano De Ambrosi, Fabrizio Faggiano

**Affiliations:** Department of Translational Medicine, University of Eastern Piedmont, Novara, Italy; Department of Translational Medicine, University of Eastern Piedmont, Novara, Italy; Department of Translational Medicine, University of Eastern Piedmont, Novara, Italy; Department for Sustainable Development and Ecological Transition, University of Eastern Piedmont, Vercelli, Italy; Department of Translational Medicine, University of Eastern Piedmont, Novara, Italy; Department of Translational Medicine, University of Eastern Piedmont, Novara, Italy; Department of Translational Medicine, University of Eastern Piedmont, Novara, Italy; Department for Sustainable Development and Ecological Transition, University of Eastern Piedmont, Vercelli, Italy; Epidemiologic Unit of the Local Health Authority of Vercelli - Osservatorio Epidemiologico, ASL Vercelli, Vercelli, Italy

## Abstract

**Background:**

The higher disease burden and related costs due to an increasing aging population have placed tremendous pressure on the healthcare systems worldwide. Given that music, both listened and actively performed, promotes and maintains good health and wellbeing among the population, we sought to perform a systematic review that would assess its biopsychosocial effects on a population over 40 years of age.

**Methods:**

A comprehensive search of peer-reviewed articles up to April 2021 was conducted on six electronic databases (i.e. Cochrane, MEDLINE, PubMed, PsycINFO, Web of Science and Scopus). Our study population only included healthy adults of 40 years and older. A total of 11 randomized controlled trials (RCTs) matched the inclusion criteria and were therefore analyzed.

**Results:**

Despite the heterogeneity of the methodologies used in the selected studies, our findings suggest that active musical participation can lead to beneficial effects on both cognitive and psychosocial functioning, whereas the positive impact of listening to music seems to be predominantly restricted to the cognitive domain.

**Conclusions:**

Although our results are consistent with both active and passive music activities favouring health and wellbeing in individuals 40 years old and over, future prospective RCTs, employing more uniformed and sensitive measurements, should allow us to better gauge the role of music participation in healthy aging and longevity, especially in countries with a high population density of elderly people.

## Introduction

A recent review by the World Health Organization^1^ highlights the important role that art, culture and music have in promoting health and wellbeing. Indeed, participation in musical events can lead to emotional, cognitive and socio-relational benefits, with a positive effect on crucial biopsychosocial functions (e.g. increased immune response, greater sense of self-efficacy, reduction of social isolation, etc.).

Research suggests that music exerts a positive effect on human health through modulation of several neurochemical systems (e.g. dopamine, opioid, etc.), thereby stimulating the perception of pleasure, reward, motivation and arousal, lessening stress and anxiety, improving the extent of social affiliation, and increasing the efficiency of the immune system.^2^ Furthermore, both active and passive music participation can improve balance and motor coordination, adherence to group physical exercise interventions^3^ and executive functions (e.g. perceptual speed, visual-scanning, verbal fluency, etc.).^4^^,^^5^ Importantly, participation in community music and singing activities can exert beneficial effects in terms of reduction of isolation, depression and mental health, especially among the elderly.^6^ Actually, different authors have shown the beneficial effects of community intervention, which improve mental health, quality of life (QOL) and social support in older people.^7^ Beyond the clinical setting, pleasure, creativity and social support promoted by music-related activities positively influence self-confidence and self-esteem, showing transversal effects among and within individuals.^8^ Exploring closely related themes, other scholars have found that music training improves social and subjective (e.g. satisfaction with life, happiness, etc.) wellbeing.^9^ Associations between playing/singing and health and health-related QOL have emerged in samples over 16, also showing that people are aware of the role of music in promoting health.^10^

In order to deepen the multidisciplinary understanding of the relationship between music and health, the objective of this study was to systematically review the available evidence on the impact of both passive (e.g. listening condition) and active (e.g. music making and choral singing) participation in music on the physical and psychosocial health of the adult population.

## Methods

This study is a systematic review of past and current literature addressing the relationship between music and health/wellbeing. It was conducted in accordance with the Preferred Reporting Items for Systematic Reviews and Meta-Analyses (PRISMA) statement.^11^

### Search strategy

A comprehensive search of published studies up to April 2021 was conducted using the following computer databases: Cochrane, MEDLINE, PubMed, PsycINFO, Web of Science and Scopus.

With regard to the keywords, we took into account both very inclusive terms referring to culture—music included—and more specific terms of the musical dimension.

Concerning the effects, we considered words related to health and wellbeing. The key terms for searches included: (‘Creativity’ OR ‘Cultural access*’ OR ‘Cultural activit*’ OR ‘Cultural participation’ OR ‘Cultural attendance’ OR ‘cultural engagement’ OR ‘cultural event*’ OR ‘cultural behavior*’ OR ‘Art* activit*’ OR ‘Art* participation’ OR ‘Art* attendance’ OR ‘Art therapy’ OR Music) AND (‘Risk factor*’ OR ‘Lifestyle behavior’ OR ‘Healthy lifestyle’ OR ‘Health*’ OR ‘health promotion’ OR ‘Health behavior*’ OR ‘Health outcome*’ OR wellbeing OR ‘Wellbeing’).

### Inclusion criteria

The original inclusion criteria for the studies were as follows: (i) healthy people aged over 40 years as target population; (ii) randomized controlled trials (RCTs); (iii) studies considering the effect of music and/or making music on health (i.e. psychophysical and social variables, health and wellbeing).

### Study selection and classification

The studies retrieved by the literature search were further selected by identifying specific keywords and concepts within titles and abstracts. Subsequently, full texts were obtained and scanned for consistency. Studies that met the inclusion criteria were categorized according to the type of music participation: either active (e.g. learning to play an instrument, choral activities, etc.) or passive (i.e. listening to music as a main activity). The duplicates were manually removed. The ultimate decision to include or exclude the articles was determined by consensus of all authors.

The considered outcomes were: (i) physical (e.g. strength, gait, etc.); (ii) cognitive (e.g. executive functions, memory, attention, etc.); (iii) affective (e.g. depression, mood, etc.); and (iv) QOL—as a separate variable due to its multi-faceted and multi-dimensional conceptualization.

### Quality evaluation of studies

The quality of the selected studies was independently assessed by two reviewers (MDV and DC) using the Critical Appraisal Skills Programme (CASP).

### Data extraction

Data useful for the review were independently extracted by two authors through close reading of the shortlisted studies and then reported on a form specifically prepared. For each outcome, the results were tabulated, taking into account their possible improvement and the differences between intervention and control groups.

### Statistical analysis

To evaluate the agreement between the two reviewers in terms of quality assessment, Cohen’s Kappa indices were calculated for each of the 11 criteria defined by CASP. In addition, the global Kappa coefficient was also considered. Finally, discordant measures were discussed and resolved.

For all studies, we considered the occurred changes over time as a result of the specific intervention. For those outcomes detected in more than two studies with homogeneous methods, we performed a meta-analysis. The mean changes in QOL parameters between the end of the study (post) and baseline (pre) in the intervention and control groups were calculated. When the standard deviation of the difference was not reported in the manuscript, the square root of variance at pre plus variance at post less 2*covariance (post–pre) was estimated using as correlation coefficient of 0.38, calculated according to Bugos’ results.^5^ When a study reported more than one intervention group, separated meta-analyses were proposed. The differences in the post–pre changes between intervention and control groups were evaluated and considered as a measure of interest for the meta-analysis.

For each outcome, the DerSimonian and Laird method was applied to calculate the random effects pooled mean difference. Heterogeneity between studies was tested using the Cochrane Q test and quantified through the *I*^2^ index.

For the other outcomes, the percentage of change over time of all the other outcomes was calculated by both intervention and control group (table 2). A synthetic indicator of effectiveness was also displayed, showing whether the result was in favour of the intervention group (+) or of the control group (–) together with the statistical significance of the difference.

**Table 2:** Main effects (*n* = 80) of the considered studies with regard to the intervention

	Outcome	Intervention	Studies	Results (% of change)		Effect
				Intervention Gr	Control Gr	
PD	Leg strength (s_ct_)**	Choral activity	Johnson	**3.31**	**–2.24**	**–**
	Gait mobility	Choral activity (postural sway_2_/ps_1_)	Johnson	–6.67	–6.67	–
		Make music (s_ct_)	Santos	**–9.26**	**–5.54**	**+**
		Listen to music (s_ct_)	Hars	**–4.12**	**–6.67**	–*
	Gait speed (m *or* cm/s)	Choral activity	Johnson	0	0	x
		Listen to music	Maclean	**–5.93 MTG**	**4.17**	**–**
				**–1.46 MPG**		
			Hars	**1.61**	**6.27**	–*
CD	Cognitive dimension (n_ca_)***	Listen to music	Borella	2.82 MG	.00	+*
				14.68 AG		
				0.77 WNG		
			Hars	3.32	6.56	–
	Verbal fluency (n_words_/s)	Make music	Bugos	**–0.40 GPI^I^**	**1.68^I^**	**–**
				**16.21 GPel^I^**		**+**
				**7.24 GPI^II^**	**1.98^II^**	**+**
				**11.85 GPel^II^**		**+**
				**12.23 GPI^III^**	**4.65^III^**	**+**
				**12.29 GPel^III^**		**+**
			Seinfeld	**–2.73**	**–10.91**	**–**
			Santos	**0.59^IV^**	**–7.36**	**+**
				**–3.40^V^**	**5.53**	**–**
		Listen to music	Borella	11.46 MG	–5.58	+*
				9.38 AG		
				–0.41 WNG		
	Attention	Choral activity (n_ca_)	Johnson	–1.37	0.00	–
		Make music	Bugos (s_ct_)	**–21.94 GPI**	**–8.65**	**+**
				**9.51 GPel**		**–**
			Seinfeld (n_ca_)	**1.85^VI^**	**0.30**	**+**
				**8.12^VII^**	**–0.20**	+*
				**7.71^VIII^**	**–4.25**	+*
				**5.12^IX^**	**–0.34**	**+**
	Planning skills/Visual spatial skills	Make music (s_ct_)	Bugos	**–17.00 GPI**	**11.14**	+*
				**–20.77 GPel**		**+**
			Seinfeld (Δ)	**–2.60**	**40.45**	**+**
			Santos^X^ (s_ct_)	**–9.23**	**–7.53**	**+**
			Santos^XI^ (n_ca_)	**24.31**	**0.44**	+*
		Choral activity (s_ct_)	Johnson	**1.49**	**1.63**	**–**
		Listen to music	Borella^XII^ (n_ca_)	11.26 MG	2.06	+
				9.24 AG		
				9.13 WNG		
			Borella^XIII^ (n_ca_)	10.98 MG	10.98	x*
				10.95 AG		
				13.15 WNG		
			Hars (n_ca_)	–6.67	–1.14	–
	Memory (n_ca_)	Choral activity	Johnson	15.79	12.73	+
		Make music	Seinfeld	**–9.69^XIV^**	**–1.79**	–*
				**–1.33^XV^**	**1.30**	**–**
			Seinfeld	**2.21^XVI^**	**13.64**	**–**
				**0^XVII^**	**1.15**	**–**
			Bugos	**7.72 GPI**	**4.68**	**+**
				**16.71 GPel**		**+**
			Santos	**0.75**	**1.13**	**–**
		Listen to music	Borella^XVIII^	**16.17 MG**	**–2.61**	+*
				**21.79 AG**		
				**12.63 WNG**		
			Borella^XIX^	**12.88 MG**	**5.43**	+*
				**8.94 AG**		
				**2.51 WNG**		
ASD	Depression (*M*)	Make music	Bugos^XX^	–4.00 GPI	–11.02	–
				–6.46 GPel		–
			Bugos^XXI^	–25.52 GPI	–35.17	–
				–44.29 GPel		+
			Yap	–42.85 GrA	–33.33 GrB	+
				–50.00 GrB	–50.00 GrA	x
			Seinfeld	–36.21	–22.02	+
		Choral activity	Coulton^XXII^	–25.45	–1.40	+
			Coulton ^XXIII^	–17.81	–9.05	+
			Johnson	**–6.98**	**–2.33**	**+**
		Listen to music	Hars ^XXIV^	–12.15	–7.92	+
	Mood (*M*)	Make music	Seinfeld	**–5.41_TOT_**	**2.51**	+*
				**–24.48_Tension_**	**4.76**	**+**
				**–19.56_Depression_**	**38.46**	**+**
				**–1.38_Vigor_**	**1.15**	**–**
				**–30.97_Fatigue_**	**49.77**	+*
				**–20.64_Anger_**	**–1.05**	**+**
				**–17.07_Confusion_**	**7.02**	**+**
		Choral activity	Johnson	–1.87_Sadness_	–1.06	+
				1.23_PositiveEmotions_	–1.56	+
				–0.99_Fear_	0.00	+
				–3.26_Loneliness_	1.01	+*
				1.10_InterestLife_	–1.09	+*
	Social dimension (*M*)	Make music	Yap	–32.43 GrA	20.00 GrB	–
				–8.33 GrB	–4.00 GrA	–
	QOL (*M*)	Make music	Yap	**16.05 GrA**	**–4.76 GrB**	**+**
				**5.00 GrB**	**–7.45 GrA**	**+**
			Seinfeld	**3.47_Physical_**	**–4.25**	+*
				**2.17_Psycological_**	**–0.98**	+*
				**4.42_Social_**	**1.01**	**+**
				**3.49_Environmental_**	**0.64**	**+**
		Choral activity	Coulton	2.05^XXV^	–0.50	+
				**7.17^XXVI^**	**–0.20**	+*
				2.63^XXVII^	1.32	+*
		Listen to music	Hars	1.59	3.57	–

Primary outcomes are in bold. PD = physical dimension; CD = cognitive dimension; ASD = affective and social dimension; QOL = quality of life; ‘Choral activity’ and ‘Make music’ = active activities; ‘Listen to music’ = passive activity; ‘+’ = in favour of intervention group; ‘–’ = in favour of control group; ‘x’ = no findings;

* *P* < 0.05;

** s_ct_ = seconds to complete the task;

*** n_ca_ = number of correct answers;

I VFT-Letter fluency;

II VFT-Category fluency;

III VFT-Category switching;

IV VFT-Phonemic;

V VFT-Semantic;

VI Stroop test—Word;

VII Stroop test—Colour;

VIII Stroop test—Word-Colour;

IX SDMT;

X TMT-A;

XI CDT;

XII MPFB;

XIII Spatial description—Map drawing;

XIV Digit span forward;

XV Digit span backward;

XVI Spatial span forward;

XVII Spatial span backward;

**XVIII:** CWMS;

XIX BC-BT;

XX GDS;

XXI BDI;

XXII HADS depression;

**XXIII:** HADS anxiety;

XXIV HADS depression and anxiety;

XXV SF12—physical;

XXVI SF12—mental;

**XXVII:** EQ5D.

All analyses were conducted using R v. 3.6.2^12^ with the metaphor library v. 2.4.^13^ Standard Mean Differences with their corresponding 95% confidence intervals (95% CI) and two-sided *P*-values were reported.

## Results

**Figure 1:**
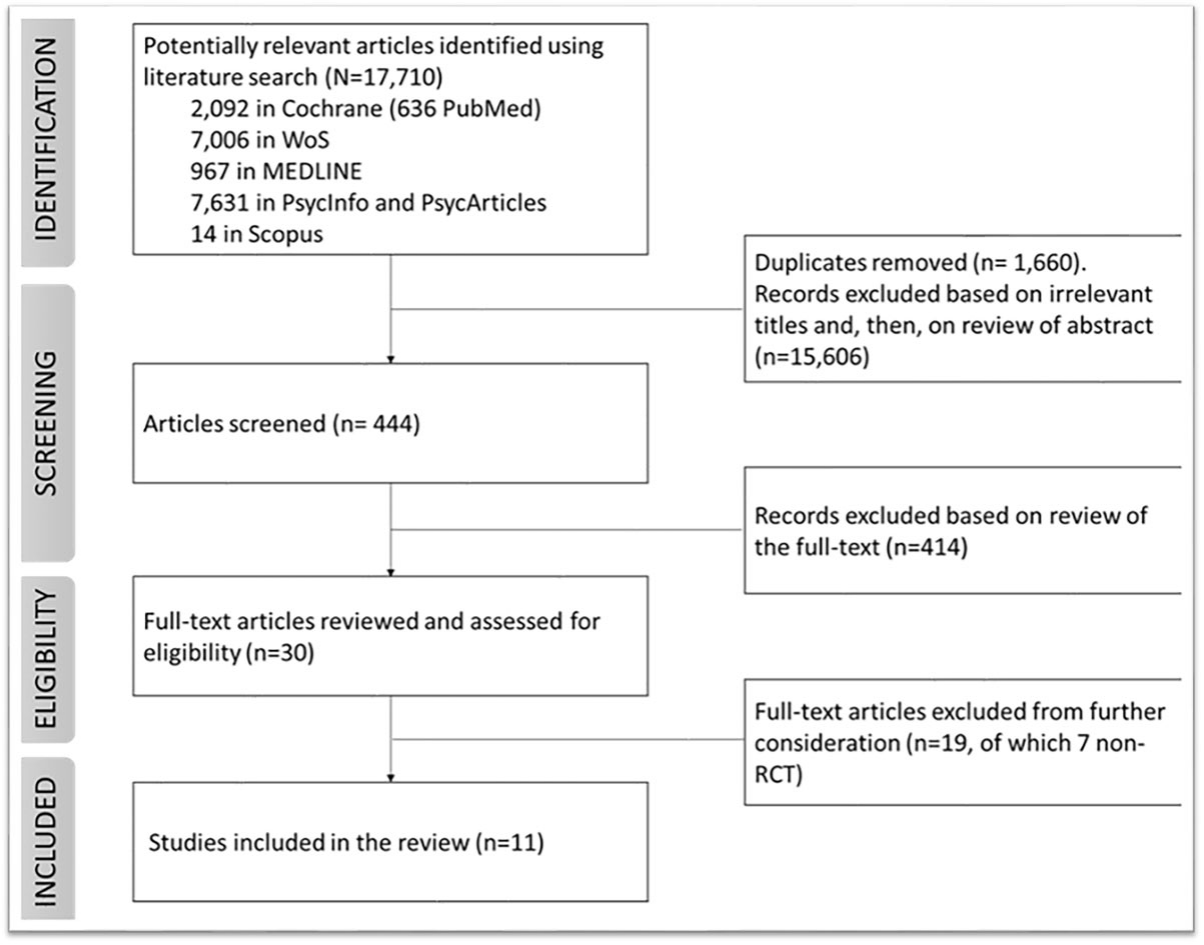
Flow chart of the literature search. Visual representation of the identification process resulting in the selection of 11 publications, included in this systematic review, from 17,710 records returned by the literature search.

After duplicate removal, our literature search returned 16 050 articles. The selection process identified 11 papers meeting the inclusion criteria, corresponding to 0.07% independent RCTs published until March 2021. Figure 1 depicts the flowchart of the identification and selection process. For details on materials and field of study, see Supplementary material S1.

### Study characteristics

The main features of the selected studies are reported in table 1. Eleven RCTs involving passive (e.g. listening to music) or active (e.g. choral singing, playing an instrument or singing) musical activities were examined in this review. In particular, three studies used a passive musical approach, focusing on physical (i.e. strength and mobility) and cognitive (i.e. executive functions and memory) effects. All other studies (*n* = 8) used active musical approaches, analyzing both physiological and psychosocial effects, also including the affective sphere and/or the holistic construct of QOL. All the considered studies analyzed the effects of music in samples of middle age and elderly people and had a time range of 2 weeks to 48 months for follow-up (*M *=* *6.9 months) except Maclean et al.,^4^ who did not consider any follow-up. The age of the participants was 49 years and older (*M *=* *71 years), the sample sizes range from 17 to 390 participants (*M *=* *102.6). The samples were not generally balanced with respect to gender—on average, samples consisted of 77.6% women (see also Supplementary material S2).

**Table 1:** Main characteristics of the considered studies with regard to the type of activity established by the intervention (passive/active or both)

First author, year	Country	*N* participants (% women)	Age (min–max)	Groups	Music education of the sample	Passive/Active participation	Outcome
Borella, 2017	Italy	72 (58%)	65–75	Four groups	No	Passive	Working memory (WM)
			Mean 69.3	− Mozart group (MG) = 19			Verbal fluency
				− Albinoni group (AG) = 19			Planning skills/visual spatial skills
				− White noise group (WNG) = 16			
				− Control group no music (NMG) = 18			
Maclean, 2013	UK	45 (62%)	65–88	Three groups	n.a.	Passive	Gait/mobility
			Mean 71.7	− Musical training group (MT) = 15			Planning skills/visual spatial skills
				− Music reproduction group (MP) = 15			Memory
				− Control group no music (NM) = 15			
Hars, 2014	Switzerland	52 (98%)	66–82	Two groups	No	Passive	Gait/mobility
			Mean 75 SD 8	− Intervention group (music-based multitasking exercise program) = 23 − Control group (no music, NM) = 29			Physical strength Cognitive dimension/executive functions Planning skills/visual spatial skills Anxiety and depression QOL
Diaz Abrahan, 2021	Argentina	Study 1: 84 (81%) Study 2: 91 (81%)	60–90 Mean (Study 1) 72.28 Mean (Study 2) 71.91	3 groups − Intervention group (free musical improvisation) − Active control group (imitation of a rhythmic pattern) − Passive control group (a period of silence)	Yes	Active	Verbal memory
Bugos, 2019	USA	135 (71%)	60–80	Three groups	No	Active	Planning skills/visual spatial skills
			Mean 68.6	− Group piano training (GPI) = 49			Memory
				− Gross motor training—Group percussion ensemble (GPeI) = 38			Verbal fluency and attention Depression
				− Music listening instruction (MLI—control group) = 38			
Coulton, 2015	UK	258 (83%)	Mean 69.2 (>60)	2 groups	n.a.	Active	Anxiety and depression
			SD 7.14	− Intervention group (singing sessions) = 131			QOL
				− Control group (normal activity) = 127			
Johnson, 2020	USA	390 (76%)	59–93	Two groups	No	Active	Physical strength
			Mean 71.3	− Intervention group (choral activity) = 208			Gait
				− Control group (no activity) = 182			Attention
							Planning skills/visual spatial skills
							Memory
							Affective and social dimensions
MacRitchie, 2020	Australia	17 (77%)	Mean 70.9 (>65)	Two groups	Yes	Active	Planning skills/visual spatial skills
			SD 5.5	− Intervention group (piano training program)			
				− Control group (waitlisted inactive)			
Santos, 2020	Brazil	28 (79%)	Mean 67.9 (>60)	Two groups	No	Active	Gait/mobility
				− Experimental group (percussion and musical improvisation) = 15			Planning skills/visual spatial skills (flexibility, inhibition, and attention)
				− Control group (participation in a choir) = 13			Cognitive dimension/executive functions
							Verbal fluency
							Memory
Seinfeld, 2013	Spain	29 (76%)	60–84	Two groups	No	Active	Attention and planning skills/visual spatial skills
			Mean 69.4	− Experimental group (piano lessons) = 13			Memory
				− Control group (different leisure activities) = 16			Affective dimension and social dimension
							QOL
Yap, 2017	Singapore	31 (93%)	68–81	− Intervention group = 16	n.a.	Active	Affective dimension and social dimension
			Mean 74.6	− Control group = 15			QOL

### Quality assessment

Overall, the agreement of quality assessors was high. Cohen’s Kappa index ranged from 0.88 to 1, whereas the overall Kappa was 0.94 (see Supplementary table S3 for Kappa statistics for each dimension of the assessment).

### Study findings

The meta-analysis could only be carried out on the ‘planning skills’ outcome, detected by TMT, which was present in three studies.

For nine studies, the percentage of improvement or worsening over time was calculated, considering both the intervention and control groups. For two studies,^14^^,^^15^ this type of analysis was not possible as the authors failed to report essential data. Therefore, the results of these two studies could only be analyzed qualitatively. Table 2 summarizes the effects of the following music-based interventions (*n* = 80): listening to music (*n* = 13); playing music (*n* = 50); and choral singing (*n* = 17). Seven results pertained to the physical sphere, whereas 38 and 35 were respectively related to the cognitive—4 results are not shown in table 2 due to missing data—and psychosocial domains. Furthermore, 65% of the effects were positive, of which 30.77% statistically significant, whereas 31.25% showed negative effects, of which 12% statistically significant. Of note, only one physical result showed a non-significant improvement (Supplementary material S4 for further comments). Of the 80 main effects (table 2), 50 effects (62.50%) are the primary outcome, of which 35 are positive (70%) and 11 (22%) are also statistically significant.

Table 2 summarizes the main effects observed in the intervention vs. control groups as reported by the aforementioned studies.

#### Physical dimension

The studies exploring the effects of music on the physical domain considered in this analysis seem to provide contrasting results and are basically negative. Given that Hars et al.^3^ found a substantial improvement in strength, gait and speed after long-term music-based multitasking training only at 4-year follow-up (but not after 1-year follow-up), it is tempting to speculate that the type and duration of intervention may yield more consistent results in terms of physical skill improvement.

#### Cognitive dimension

**Figure 2:**
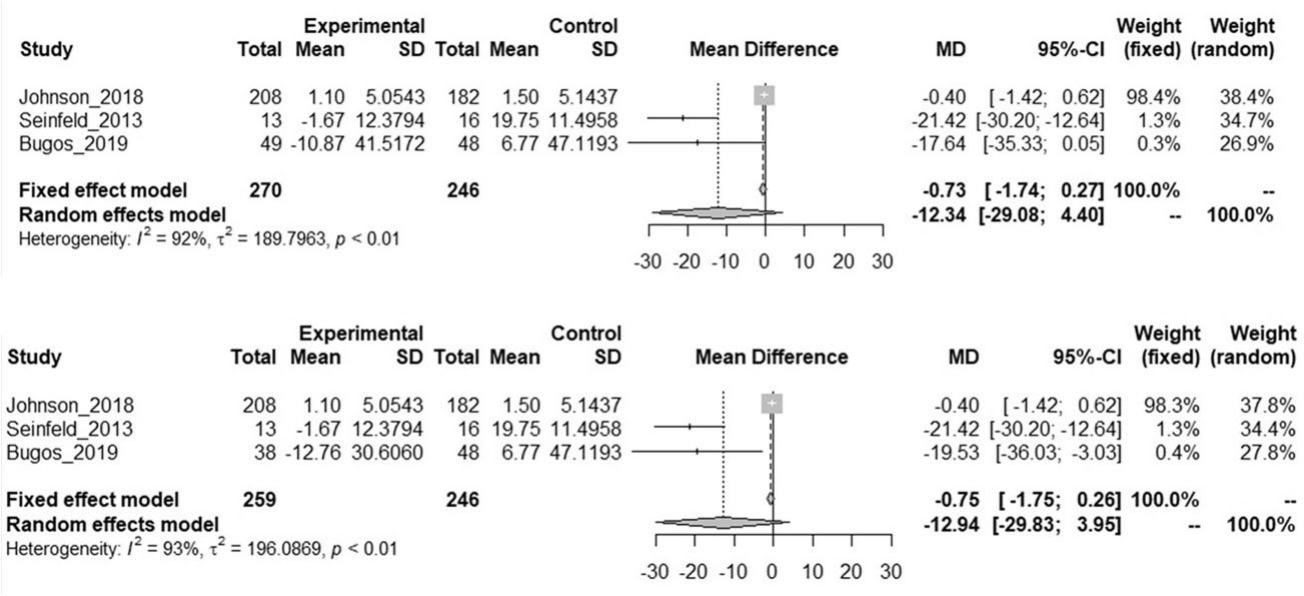
Meta-analysis on post-pre TMT difference in experimental and control group. Mean differences are reported with 95% CI. In the upper panel, we chose piano-trained participants as the intervention group, while in the lower panel the intervention group consisted of percussion-trained subjects (Bugos, 2019).

Concerning the findings on cognitive dimension and executive functions, only two studies did not consider the effects of music on the cognitive sphere. Twenty-four out of 38 results are positive effects, and 9 are also statistically significant. As for the effects at the physical level, these results are neither univocal nor easy to generalize. Purely listening to music seems to have a positive effect on fluid intelligence, verbal fluency, spatial skills and memory. Most of the studies mainly consider executive functions—particularly through TMT—and memory—using different tools—showing a particularly positive effect on memory. Above all, the activity of playing music appears to be more effective in terms of memory, attention and executive functions. The paucity of studies on verbal fluency and spatial skills do not allow us to infer significant effects. Based on the results from the articles by Johnson et al.,^16^ Seinfeld et al.^17^ and Bugos,^5^ we performed meta-analysis to assess TMT differences in post and pre measurements between intervention and control group. Mean differences were respectively –12.34 (95% CI: –29.08 to 4.40] and –12.83 (95% CI: –29.83 to 3.95). The forest plots are depicted in figure 2.

#### Affective and social dimension

The results on the psychological domain and QOL from six studies show various degrees of improvement in the intervention groups (27/35), with eight of them statistically significant. Once more, the heterogeneity of the measures does not allow us to draw a definite conclusion. Exchanging the intervention group (GrA) with the control group (GrB) from phase 1 to phase 2 of their study, Yap et al.^18^ did not observe statistically significant differences neither on depression nor on QOL (with positive trend) nor at the social network level (with negative trend). Despite the similarity of the interventions and the use of the same measurements to detect depression, Bugos^5^ found no significant effects, whereas Seinfeld did. With different measurements and interventions, Seinfeld et al.^17^ and Coulton et al.^19^ reported significant results in terms of depression and anxiety (Coulton) reduction, mood improvement (Seinfeld) and QOL. A more detailed description of the results is provided in Supplementary material S4.

### General considerations about the measures

Given the heterogeneity of the measures used in the selected studies, this section aims to provide a reflection on the measures that, according to our analysis, seem to be more sensitive and suitable for empirical studies. The effects of similar interventions on the cognitive dimension measured by MMSE suggest that this tool represents an excellent screening test for cognitive impairment. On the other hand, the results of the interventions on verbal fluency indicate that FLT and VFT may sometimes lead to discordant values and that they may not be sensitive enough to predict considerable effects. Overall, the Stroop Test for the evaluation of attentional processes seems to be a more sensitive tool than others—Seinfeld et al.^17^ reported a lower sensitivity of SDMT than that of the Stroop Test, with the latter showing more robust effects. With respect to the executive functions, TMT does not seem to be a particularly functional and sensitive tool. Indeed, Santos et al.^20^ reported significant results only when using CDT but not TMT. Other tools that seem adequate are MPFB and map drawing. Concerning the memory dimension, digit span—rather than spatial span, which in the same study by Seinfeld et al. does not show significant results—Corsi task and CWNS appear to be reliable and sensitive tools. Finally, concerning the affective dimension, measures of depression or anxiety do not lead to significant results. Conversely, multidimensional measures related to mood and QOL provide quite substantial results, especially in terms of discriminating the dimensions most influenced by the proposed intervention. Concerning the physical measures, we cannot draw any firm conclusions due to the strong heterogeneity of the studies taken into account.

## Discussion

Whereas participation in music-related activities does not result in significant improvements at the physical level, it seems to produce positive effects on most of the cognitive dimensions analyzed, especially attention. Similar beneficial effects are found at the psychosocial level, where music participation can reduce the symptoms of depression and anxiety, thereby improving QOL.

The impact of both active and passive musical activities on the physical dimension is probably negligible, with at least two studies suggesting a tendency to worsen. This is, however, in contradiction with a study showing that long-term (4 years’ follow-up) music-based multitasking exercise is effective in preventing age-related physical decline and falls in older adults,^3^ raising the attractive hypothesis that integrating listening to music with physical activity may be an effective physical strategy. Choral singing does not seem to have any impact on gait and balance.^16^

Regarding the cognitive dimension, the two interventions ‘music making’ and ‘listening to music’ mostly act on verbal fluency, attention, planning skills and memory. Specifically, playing an instrument seems to prevent the cognitive decline caused by aging.^20^ Similarly, listening to music, albeit to a lesser extent, seems a promising strategy to prevent cognitive decline. Unfortunately, the scarcity of the results concerning choral singing interventions do not allow us to draw any conclusion.

Most results show a positive impact of music participation on the affective dimension, with no differences between active and passive activities. In particular, playing the piano and learning to read music appear to improve both cognitive abilities and subjective wellbeing.^17^ No substantial results are available for depression and anxiety, probably due to the predominance of healthy people, who generally have fewer mental problems, among the participants. Consequently, focusing on more specific dimensions of psychosocial wellbeing may be a better approach to grasp the subtler processes through which music acts over time.

Concerning the psychological aspects and QOL, Coulton et al.^19^ show that active music participation based on singing exerts beneficial effects. In good agreement, Johnson et al.,^16^ report the positive impact of choral singing on loneliness and interest in life, despite the absence of significant effects on depression. Finally, playing an instrument and singing improves the multidimensional variable of QOL. The main effects associated with the primary outcomes of the studies that showed statistically significant improvements were all associated with cognitive, affective and quality-of-life dimensions, none of these were associated with any physical dimension.

The results from this review concur with previous studies supporting the important role of music participation in promoting both cognitive and affective wellbeing. In particular, active music participation appears to be a crucial intervention to improve both cognitive and emotional dimension, whereas listening to music seems to produce better cognitive effects. However, this study has an important limitation due to the heterogeneity of the methods adopted by the selected studies, which has prevented—and will prevent—the generation of robust meta-analyses. Consequently, the results are often discordant, and the effects of the interventions appear rather weak, thus making it difficult to outline effective guidelines for musical interventions aimed at wellbeing and also due to the heterogeneity of the methodology of the study it is difficult to identify which one is the primary outcome. In light of these considerations, it is recommended that future research should try to homogenize the methods, using more sensitive and appropriate measures for the studies, thus ensuring a greater result comparability.

Several studies support our findings. For example, Daykin et al.^6^ showed the positive impact of music and singing on wellbeing, reducing or preventing depression in adults across their lifespan. Moreover, a review by Sheppard and Broughton,^21^ reported that active music participation is effective in maintaining and promoting well-being and health throughout life. According to these authors, also in light of the aforementioned limitations of the studies analyzed in this review, there are some limitations that need to be addressed in future research. In addition to the heterogeneity of the methods, many of the samples analyzed are too small, thereby limiting the generalizability of the results. Furthermore, as indicated by Daykin et al.,^6^ the socio-demographic factors (i.e. gender and ethnicity) that could influence the relationship between musical participation and wellbeing should also be considered. The studies tend to consider specific age groups; our review, while wishing to focus on the over 40s, was able to select studies that considered at most the over 59s. To some extent, this limits the in-depth understanding of the role of music in adults in anticipation of a healthy and active aging. Moreover, it is necessary to align the measures in order to allow future integrated analyses and make proper comparisons, understanding the deeper effects of the proposed interventions. Likewise, it is imperative that researchers use the most sensitive measures to detect variables while considering their appropriateness in relation to the sample being analyzed. Furthermore, considering wider age groups, future studies will have to use (especially for physiological variables) adequate tools in order to detect the real effects. Furthermore, it is plausible that music education has a role in the relationship between music and health/wellbeing, this aspect should be reported and taken into account in every study, but this information is not present in all of the study of the review, so it is important that future research must investigate this dimension.

Finally, given its complexity, the relationship between musical participation and wellbeing should be addressed in more transversal academic fields, thus contributing to a relatively unexplored topic in both health and cultural sectors. Indeed, our analysis of the first authors and publication journals reveals that the selected studies were published by researchers mainly from the medical neuroscience field. Thus, it is recommended that researchers cooperate with both close (e.g. psychology) and distant (e.g. arts, socio-political) fields of study in order to devise more effective music interventions in health promotion.

## Conclusion

The results of this systematic review suggest that active and passive music activities can positively influence health and wellbeing. Although common sense considers music as an effective treatment, the data considered herein are not robust enough to draw valid conclusions on the effectiveness of music participation. Consequently, we strongly believe that it is time to conduct one or more prospective RCTs able to confirm this suggestion, overcoming the limitations found in the studies analyzed. To this end, these clinical trials should involve adults partially involved in music, be randomized and adequately powered, and adopt more valid scales—according to our findings—to measure the outcomes. Moreover, they should possibly evaluate biomarkers of chronic inflammation and oxidative stress in order to determine, with consistent methods, whether music participation can influence physiological responses. Finally, we stress the importance of increasing our understanding of music-driven biopsychosocial effects in countries with a high population density of individuals over 40 years of age (e.g. Italy = 60.7%, Spain = 58.8%)^22^ that more than others could benefit from music-based interventions to promote healthy aging and longevity.

## Supplementary Material

ckad063_Supplementary_Data

## Data Availability

Data availability is not applicable to this article as no new data were created or analyzed in this study. Key points The studies evaluated in this review show the crucial role of music in health promotion in middle and older age. Participation in music-related activities does not result in significant improvements at the physical level. Active musical participation can improve both cognitive and psychosocial functioning; passive musical participation can mostly improve the cognitive domain.
