# Supplementary material for: The role of music in promoting health and wellbeing: a systematic review and meta-analysis
Source: Eur J Public Health. 2023 Jun 15;33(4):738–45. doi: 10.1093/eurpub/ckad063 (PMC10393487; doi:10.1093/eurpub/ckad063)
Supplement: ckad063_Supplementary_Data [file ckad063_supplementary_data.zip › ckad063_Supplementary_Data/Supplementary Document S2.docx]

**Supplementary material S2**

**Study characteristics: further information**

Concerning the studies based on passive intervention (n = 3), the authors specifically considered the effects of listening to music on the following functions: gait mobility (n = 1), speed cognition (n = 2), verbal fluency, planning skills and short- and long-term memory;^23^ gait stability and cognitive functioning^4^—this study assessed the effects of rhythmic music training on participants instructed to walk according to the rhythm while listening to music—; and physical function and risk of falling^3^—this study evaluated the effects of music-based multitasking exercises on the participants’ motor skills. The first two studies used gender-balanced samples, while the third one was characterized by an almost total prevalence of women. Borella et al.^23^ considered a 6-month follow-up, whereas Hars et al.^3^ had two follow-ups at 1 and 4 years.

Eight studies considered the effects of active participation in music. The following 5 studies considered the act of playing instruments: Diaz Abrahan et al.^14^ observed the effects of musical improvisation, considering both musicians and non-musicians in late-life; Bugos^5^ assessed the effects of musical training in either piano—similarly to Seinfeld et al.^17^ and MacRitchie et al.^15^—or percussions; Yap et al.^18^ developed a rhythm-centered music making intervention.

Two studies—Coulton et al.^19^ and Johnson et al.^16^—examined the impact of community group singing (i.e., choir intervention). Furthermore, considering both the activity of playing an instrument and singing, Santos et al.^20^ compared the outcome of a percussion-based intervention and musical improvisation to that of a choir activity.

In general, all the authors used different tools to measure the same variables (e.g., gait detection through Gait-usual gait speed or the NIH Toolbox Performance Measures) (see Appendix B).

According to their protocols, two studies scheduled their follow-ups at 6 months, one study at 4 months, whereas the remainders had them planned at around 2.5 months. All studies considered gender-biased samples, with a greater presence of women.
