# Supplementary material for: The role of music in promoting health and wellbeing: a systematic review and meta-analysis
Source: Eur J Public Health. 2023 Jun 15;33(4):738–45. doi: 10.1093/eurpub/ckad063 (PMC10393487; doi:10.1093/eurpub/ckad063)
Supplement: ckad063_Supplementary_Data [file ckad063_supplementary_data.zip › ckad063_Supplementary_Data/Supplementary Document S4.docx]

**Supplementary material S4**

**Study findings: further comments**

5 physical results showed deterioration, with 2 results being statistically significant. With regard to the results related to the cognitive domain, 63.16% of them showed an improvement (33.33% statistically significant). 34.21% of cognitive results displayed worsening signs (7.69% statistically significant). Concerning the psychosocial effects, 77.14% of them were positive (29.63% statistically significant). Finally, 20% of the psychosocial effects showed a negative trend (no statistically significant results).

Considering the types of intervention, 13 effects are related to listening to music, 6 of which showed an improvement (4 statistically significant) and 6 a deterioration (2 statistically significant; another effect does not show any changes). 50 studies reported results related to making music, 34 of which showed an uptrend (8 statistically significant) and 15 worsening signs (1 statistically significant; another effect does not show any changes). Lastly, 17 studies reported results related to choral singing, 14 of which showed an amelioration (4 statistically significant) and 4 a decline (another effect does not show any changes).

Concerning the percentages of the results of the three types of interventions, we observed that, excluding listening to music, health improvement far exceeded health worsening over time (68% for making music, 82.35% for choral singing).

Regarding the study of Hars et al.,^3^ we consider the results concerning the single-task “walking at a speed comfortable for the participant” at the one-year follow-up. We chose to analyze only this outcome at one-year follow-up to ensure homogeneity across studies. Indeed, they considered 2 follow-ups at 1 and 4 years and, besides the aforementioned function, they also analyzed the task “walking while counting aloud backwards”, which will be discussed separately.

**1. Physical dimension**

**1.1. Leg strength**

*Active participation*

Johnson et al.^16^ reported no significant effects of the intervention on lower body strength.

*Passive participation*

From 1 to 4-year follow-up (data not shown in Table 2), Hars et al.^3^ showed that participants in the intervention group had maintained their performance in terms of functional muscle performance (strength/power) (*M*t_1_ and *M*t_2_ = 11.8), whereas the controls showed a decrease in functional lower extremity strength (*M*t_1_ = 12.3 and *M*t_2_ = 14.7) (*p* < 0.001).

**1.2. Gait mobility**

*Active participation*

Johnson et al.^16^ and Santos et al.^20^ did not find any statistically significant differences between their respective interventions and measures. However, Santos et al.^20^ showed a slight improvement in favor of the intervention group.

*Passive participation*

Though Hars et al.^3^ showed a greater improvement in the control group compared to the intervention group at one year—which led us to consider this effect as negative—, at 4 years they found a worsening trend in either group, especially the control one (*p* < 0.001).

**1.3. Gait speed**

*Passive participation*

While Maclean et al.^4^ found a non-statistically significant deterioration in performance, Hars et al.^3^ showed a significant decline. However, when Hars et al.^3^ assessed gait speed under single-task condition at 1 and 4-year follow-up, he found a significant improvement in the intervention group compared to control, with this latter showing deterioration in all tasks in terms of velocity, stride length and cadence (*p* < 0.05, for all changes). Under dual-task condition, similar results were obtained for velocity (*p* = 0.01). In contrast, stride length of the trained participants remained almost unchanged from baseline, while that of the control group worsened (*p* = 0.01). No significant effect was seen on cadence. From baseline to 4-years, velocity and stride length were the only tasks showing statistically significant differences (*p* = 0.006 and *p* = 0.001, respectively), with a substantial improvement in the intervention vs control group. Concerning gait variability (i.e., stride time and length variability, step time and length variability), under single-task condition from 1 to 4-years, a significant improvement in the intervention group emerged only in terms of step length variability (*p* = 0.003; and *p* < 0.05, for all the other variations). Considering the long-term changes from baseline to 4 years, we noticed a general and significant maintenance of the performance in the intervention vs control group, with the latter showing a worsening trend (*p* < 0.05, for all the changes). Under dual-task condition, there were no significant changes, with the exception of stride and step length variability from 1- to 4-years (*p* = 0.02 and *p* = 0.01, respectively), which showed an improvement in the intervention group and a deterioration in the control group. Concerning the balance, the only significant change emerged in terms of maintenance, showing a progressive improvement in the intervention group (*p* = 0.01).

**2. Cognitive dimension/Executive functions**

*Passive participation*

As for the analysis of fluid intelligence (i.e., reasoning skills), Borella et al.^23^ showed a significant improvement among trained participants compared to control (*p* < 0.05), whereas Hars et al.^3^ found no significant improvement in the entire sample over time. Specifically, at baseline the control group showed a Mini-Mental State Examination (MMSE) score of 25.9 (intervention group = 27.1), whereas at four years it reached 26.9 (intervention group = 27.4), barely below the cut-off point, set at 24.

**2.1 Verbal fluency**

*Active participation*

Even though Bugos^5^ showed a significant change over time for the dimensions of category fluency and category switching (*p* < 0.01, for both categories), the interaction Group × Time showed no significant results (Table 2). Seinfeld et al. (2013) as well as Santos et al.^20^ found no significant difference in verbal fluency tasks (VFT) in terms of both phonetic and semantic dimensions. However, they noted an improvement in phonetic task in the intervention group.

*Passive participation*

Concerning Borella and colleagues’ study,^23^ the intervention group showed a better performance over time compared with the control group (t_1_ and t_2_ *p*s < 0.01).

**2.2 Attention**

*Active participation*

Using the NIH Toolbox Flanker Inhibitory Control and Attention Test, Johnson et al.^16^ found no statistically significant effect in terms of attention. Through the Cued Color Word Stroop, Bugos^5^ did not find any significant effects on response inhibition for Group × Time interaction with repeated-measure ANOVA, although an improvement in performance emerged for the piano-trained group. Seinfeld et al.^17^ found a significant difference between groups (*p* = 0.03) and with respect to the Group × Condition interaction (*p* = 0.03) by symbol coding (Stroop-Color; SC) sub-test; over time, the intervention group showed an improvement in task execution. The results also showed an effect in the Group × Condition interaction for the Stroop Color-Word (SCW) sub-test concerning executive function and cognitive inhibition (*p* = 0.04), showing an improvement in the intervention group and a deterioration in the control one. No significant effect emerged upon Stroop-Word (SW) sub-test. No significant effect emerged in terms of sustained attention—assessed by Symbol Digit Modalities Test (SDMT)—as well as of problem solving strategies (Formal Lexical Task).

**2.3 Planning skills / Visual spatial skills**

*Active participation*

Bugos^5^ found no change over time in visual scanning/working memory (TMT Delta - Δ), whereas she found a significant Group × Time interaction for the same variable (*p* = 0.03) with an improvement in performance for GPI compared to MLI (Table 2). With regard to the TMT-A, Seinfeld and colleagues,^17^ reported that the piano-trained group completed the task faster in the post-vs pre-program compared to the control group. No significant effect was found for TMT-B; TMT-Δ showed a non-significant improvement.

Santos et al.^20^ found that improvised musical activities had an impact on planning and inhibition functions. In particular, visuospatial and planning abilities—assessed by Clock-Drawing Tests (CDT)—were significantly improved in the experimental group, with an increase in CDT score by 1.33 (*p* < 0.05, Bonferroni Test), whereas in the control group there was a non-significant decrease in performance (the decrease was .69, *p* = ns, Bonferroni Test). Regardless of the group, the results showed gains concerning sustained attention (TMT-A) in favor of the intervention group. Due to the reported difficulties of the participants in the correlated task (TMT-B), the authors did not analyze these latter results. Johnson et al.^16^ showed no significant Group x Time differences after 6 months concerning executive functions. MacRitchie et al.,^15^ whose results are not reported in Table 2 as they could not be analyzed, showed a moderate improvement in TMT-A and a worsening of more challenging executive functions (TMT-B as well as TMT- Δ).

*Passive participation*

Borella et al.2^3^ showed no significant effects in passive participation skills measured by Minnesota Paper Form Board (MPFB). Nonetheless, the results showed that for groups listening to structured music (MG and AG) or a non-specific sound (WNG) the score increased by one point (slightly less for the WNG group) with respect to a slight increase by 0.22 in the control group. Concerning the spatial description task, a significant main group effect emerged (*p* < 0.001) for all the three intervention groups only at post-test (no significant effects at follow-up). The results reported by Hars et al.^3^ showed no effect over time or any difference between intervention and control groups, with the abilities of the participants in both groups remaining stable over time.

**2.4 Memory**

*Active participation*

Abrahan et al.,^14^ whose results could not be summarized in Table 2, found improved verbal memory regarding the acquisition of neutral verbal information (Study 1) in the intervention vs control group. This was true for both the deferred free-recall task (main effect of the intervention, *p* < 0.001) and the deferred memory task of recognition (*p* = 0.006). In addition, the participants with musical experience performed better than non-musicians (main effect of training, *p* = 0.03). Concerning emotional verbal information (Study 2), the same differences emerged between musicians and non-musicians irrespective of memory task (*p* < 0.01). No differences were found regardless of the type of intervention. Using repeated-measure ANOVA of Digit Span Forward (DSF) scores, Seinfeld et al.^17^ showed a significant worsening over time in the intervention group (*p* = 0.023), whereas no change emerged in the control group. Although the post-test scores fell within normal range, it should be pointed out that the DSF was the only measure with significant differences between the groups at baseline in favor of the experimental group. No significant effects were found for both Digit Span Backward (DSB) and Spatial Span Backward (SSB). As shown in Table 2, Bugos^5^ found a positive change over time concerning working memory and attention (measured through PASAT; *p* < 0.001) as well as an improvement trend for the GPI group (*p* = 0.02). However, no significant Group × Time differences emerged for processing speed. Santos et al.^20^ found no statistically significant differences between improvisation group and choir.

*Passive participation*

Borella et al.^23^ showed that the three trained groups performed better than the control group ay both post-test and follow-up (*p*s = 0.001); the WNG differed from the other two trained groups (post-test *p* = 0.05, follow-up *p* = 0.03); AG performed better than MG, but only at post-test (*p* = 0.005 and *p* = 0.06 at follow-up). In contrast, concerning the backward Corsi blocks task, the authors did not find significant effect in the trained vs control group at both t_1_ and t_2_. Maclean et al.^4^ did not find improvement in working memory and general cognitive performance in all their groups (data not shown in Table 2).

**3. Affective dimension and social dimension**

*Active participation*

Bugos^5^ found a non-significant reduction of depression using Beck Depression Inventory (BDI) and Geriatric Depression Scale (GDS). Particularly, the group percussion ensemble (GPel) showed the greatest improvement, whereas the group piano training (GPI) showed the lowest improvement. Yap et al.^18^ also found no significant effect of rhythm-centered music making on depression (GDS). These studies also indicate a reduction in depression levels over time, both in the intervention and control group. They also found non-significant worsening in social isolation level, mostly in the intervention group. Seinfeld and colleagues’^17^ study showed a decrease in depression levels over time in both groups considered (Experimental group: *M*t_0_ = 8.92 and *M*t_1_ = 5.69; Control group: *M*t_0_ = 7.13 and *M*t_1_ = 5.56), with a significant main effect emerging in favor of the experimental group (*p* = 0.012). They also evaluated the impact of music on mood^[[1]](#footnote-1)^. The results showed a significant Group × Condition interaction in the fatigue dimension (Experimental group: *M*t_0_ = 4.23, *M*t_1_ = 2.92; Control group: *M*t_0_ = 2.13, *M*t_1_ = 3.19; *p* = 0.015) and in the total score of the scale (EG: *M*t_0_ = 117.70, *M*t_1_ = 111.33; CG: *M*t_0_ = 104.31, *M*t_1_ = 106.93; *p* = 0.04). Except for the dimension of anger, in which an improvement was observed in both groups, albeit more robust in the intervention vs control group, for each dimension considered there was an improvement in the intervention group accompanied by an opposite trend in the control group. Johnson et al.^16^ found a non-significant reduction in depression level at six-month in both the intervention (*M*t_0_ = 4.3; *M*t_1_ = 4) and control group (*M*t_0_ = 4.3; *M*t_1_ = 4.2). Even though there was no significant effect with regard to the Group x Time interaction, a significative improvement emerged in the subscales of loneliness and interest in life (*p* = 0.02 and *p* = 0.008, respectively). Particularly, in the intervention group, loneliness decreased (*M*t_0_ = 52.1; *M*t_1_ = 50.4), whilst interest in life increased (*M*t_0_ = 72.6; *M*t_1_ = 73.4) over time. Opposite results were observed in the control group (*M*t_0_ = 49.6; *M*t_1_ = 50.1; *M*t_0_ = 73.1; *M*t_1_ = 72.3, respectively). Coulton et al.^19^ did not find statistical significant differences at 6 months for both depression and anxiety. However, statistically significant improvement at 3 months emerged for both depression (intervention group: *M*t_0_ = 4.95, *M*t_1_ = 2.63; control group: *M*t_0_ = 4.28, *M*t_1_ = 4.15, with a mean difference between the results = -1.52 and *p* < 0.01) and anxiety (intervention group: *M*t_0_ = 6.40, *M*t_1_ = 4.14; control group: *M*t_0_ = 6.41, *M*t_1_ = 6.01, with a mean difference between the results = -1.78 and *p* < 0.01).

*Passive participation*

Hars and colleagues’ results showed no statistically significant effects over time or differences between the intervention and control groups.^3^ However, the levels of anxiety and depression had a tendency to decrease in both groups.

**4. QOL**

*Active participation*

Yap et al.^18^ did not find a significant improvement—despite the positive trend—in QOL in both groups at post-intervention (median: group A = 0.81 pre- and 0.94 post-intervention; group B = 0.60 pre- and 0.63 post-intervention). The generalized linear regression model also showed no significant improvement in QOL scores by both analyzing the change of this variable as a continuous measure and performing binary analysis. Concerning their primary outcome, Coulton et al.^19^ showed an improvement in mental health-related QOL at 6 months for the intervention group (means intervention group: t_0_ = 48.8, t_2_ = 52.3; control group: t_0_ = 50, t_2_ = 49.9), with a mean difference between the results of 2.35 (*p* = 0.05). The same trend could be observed at 3 months for the secondary outcome (IG: t_1_ = 55.5; CG: t_1_ = 50.7), with a mean difference of 4.77 (*p* < 0.01). No statistically significant differences emerged in the physical sphere of health-associated QOL at both 6 and 3 months. Concerning the different dimensions of QOL, Seinfeld et al.^17^ reported a significant Group × Condition interaction for physical health (*p* = 0.015) and psychological health (*p* = 0.045). Both dimensions showed improvements in the experimental group (physical health: *M*t_0_ = 28.85 and *M*t_1_ = 29.85; psychological health: *M*t_0_ = 21.61 and *M*t_1_ = 22.08) and an opposite trend or no changes in the control group (physical health: *M*t_0_ = 30.81 and *M*t_1_ = 29.50; psychological health: *M*t_0_ = 23.50 and *M*t_1_ = 23.27). No significant effects related to the social and environmental health dimensions were detected.

*Passive participation*

Hars and colleagues’ results showed no statistically significant effects over time or differences between groups: wellbeing levels remained almost stable in the entire sample from baseline to the last follow-up.^3^

1. We decided to discuss these findings in this section because they are related to the affective sphere. [↑](#footnote-ref-1)
